# Identification of induced mutations in hexaploid wheat genome using exome capture assay

**DOI:** 10.1371/journal.pone.0201918

**Published:** 2018-08-13

**Authors:** Momina Hussain, Muhammad Atif Iqbal, Bradley J. Till, Mehboob-ur- Rahman

**Affiliations:** 1 Plant Genomics & Mol. Breeding Lab, National Institute for Biotechnology & Genetic Engineering (NIBGE), Faisalabad, Pakistan; 2 Department of Biotechnology, Pakistan Institute of Engineering and Applied Sciences (PIEAS), Nilore, Islamabad, Pakistan; 3 University of Vienna, Department fürChromosomenbiologie, Vienna, Austria; Institute of Genetics and Developmental Biology Chinese Academy of Sciences, CHINA

## Abstract

Wheat is a staple food crop of many countries. Improving resilience to biotic and abiotic stresses remain key breeding targets. Among these, rust diseases are the most detrimental in terms of depressing wheat production. In the present study, chemical mutagenesis was used to induce mutations in the wheat variety NN-Gandum-1. This cultivar is moderately resistant to leaf and yellow rust. The aim of mutagenesis was to improve resistance to the disease as well as to study function of genes conferring resistance to the disease. In the present investigation, a 0.8% EMS dose was found optimum for supporting 45–55% germination of NN-Gandum-1. A total of 3,634 M_2_ fertile plants were produced from each of the M_1_ plant. Out of these, 33 (0.91%) and 20 plants (0.55%) showed absolute resistance to leaf and yellow rust, respectively. While 126 (3.46%) and 127 plants (3.49%) exhibited high susceptibility to the leaf and yellow rust, respectively. In the M_4_ generation, a total of 11 M_4_ lines (nine absolute resistant and two highly susceptible) and one wild type were selected for NGS-based exome capture assay. A total of 104,779 SNPs were identified that were randomly distributed throughout the wheat sub genomes (A, B and D). Induced mutations in intronic sequences predominated. The highest total number of SNPs detected in this assay were mapped to chr.2B (14,273 SNPs), which contains the highest number of targeted base pairs in the assay. The average mutation density across all regions interrogated was estimated to be one mutation per 20.91 Mb. The highest mutation frequency was found in chr.2D (1/11.7 kb) and the lowest in chr.7D (1/353.4 kb). Out of the detected mutations, 101 SNPs were filtered using analysis criteria aimed to enrich for mutations that may affect gene function. Out of these, one putative SNP detected in *Lr21* were selected for further analysis. The SNP identified in chimeric allele (*Lr21*) of a resistant mutant (N1-252) was located in a NBS domain of chr.1BS at 3.4 Mb position. Through computational analysis, it was demonstrated that this identified SNP causes a substitution of glutamic acid with alanine, resulting in a predicted altered protein structure. This mutation, therefore, is a candidate for contributing to the resistance phenotype in the mutant line. Based on this work, we conclude that the wheat mutant resource developed is useful as a source of novel genetic variation for forward-genetic screens and also as a useful tool for gaining insights into the important biological circuits of different traits of complex genomes like wheat.

## Introduction

Wheat has remained as an important part of human diet providing energy and protein since the dawn of human civilization. Generally over the course of history, wheat has been grown on 222.16 million h^-1^ with 752 million metric tons annual production worldwide (https://apps.fas.usda.gov/psdonline/circulars/production.pdf). Out of the cultivated wheat species, *Triticum aestivum* L. (allohexaploid, AABBDD genome), grown on more than 90% area, originated ~10,000 years ago by the hybridization of tetraploid wheat with *Aegilops tauschii* (diploid, DD genome) [1]. Its genome size (~17.6 GB) is relatively larger than that of many cultivated important food crops [2].

Large efforts are made to improve and/or sustain wheat production by developing varieties with more advanced genetics. However, there are still many factors which create concerns for the sustainability of wheat production (Tester and Langridge, 2010). Among these, rust diseases are one of the most vital challenges for many parts of the world, including Pakistan. Among the rust diseases, leaf rust and stripe rust are the most prevalent diseases in Pakistan. Stem rust, though not currently present, can be considered a potential threat to wheat in Pakistan[3]. All these rust diseases can substantially reduce the worldwide wheat production, and losses range from 10–70% for yellow rust, 30% for leaf rust and 50% for stem rust. Over the last few years, infection by leaf and/or stripe rust has been reported every year with varying degree of damage. This situation is alarming for the policy makers as it is raising issues surrounding food security.

Several efforts are made to counter the rust diseases, however, breeding to combat rust diseases is made difficult by the narrow genetic base of wheat cultivars, changing virulence profile of the pathogen, and the lack of sufficient genetic and genomic knowledge of the resistance reaction [4–7]. Multiple strategies including screening of wheat germplasm in disease nurseries, introgression of resistant genes from its wild species [8] and generation of mutant resistant material, have been adopted to overcome the issue. Creation of novel genetic variations in wheat genomes through inducing mutation(s) by chemicals or radiation is one alternative. In conventional mutation based strategies can be inefficient owing to variations in mutagenesis that are not easily estimated phenotypically.

With the advent of genomic tools, mutations in a genome can be identified using conventional fingerprinting approaches and next generation sequencing (NGS) assays. The ploidy level together with large (around 17 GB) and complex nature of wheat genome makes the process of re-sequencing difficult [9]. Most genes are present in multiple functional copies. This hinders the ability to unambiguously assign identified mutations to one of the homologs [10]. As with other polyploid species, the wheat genome can tolerate a high number of accumulated mutations without any significant impact on survival of wheat plant [11–12]. Interestingly, the frequency of mutations induced by EMS in the hexaploid wheat genome is almost 10 times higher than that of diploid barley genome [13].

Whole genome sequencing assays are being used extensively to characterize the genotypic diversity in mutants; however, re-sequencing cost of wheat genome is still high owing to its large genome size and the requirement of higher coverage for calling single nucleotide variation [14]. To addresses this, a subset of the genome can be enriched and sequenced. For example, one can predict that many useful mutations will be present in genes. The whole complement of exons can be sequenced using an exome capture approach [15]. This results in a dramatic reduction in the number of bases sequenced (more than 100-fold for wheat), leading to lower assay costs. Most SNP discovery initiatives in wheat explored the coding (exons) regions for identifying SNPs [16]. Further, mapping and mutation calling are made easier as repetitive regions of the genome can be avoided.

Exome capture assays have been extensively used to detect variants involved in conferring important traits in a number of crop species. For example, genetic diversity and variations in wheat and barley genomes are explored[17]. Similarly, exome capture is used to tag variations in genes conferring biomass production in corn [18]. In another study, mutations responsible for woody traits in Eucalyptus exome are identified [19]. Further studies documented the efficacy of this technology to associate variations with phenotypic diversity in black cottonwood [20], wheat [21, 22], barley [17] and switchgrass [23]. This procedure is also used to detect deletion mutations induced through radiations in soybean [24]. Thus, the function of the genes can be identified by exploring exomes in mutant populations developed by exposing the seed with chemicals or radiations [25, 26].

Before initiating re-sequencing or exome capture assays, it is important to phenotypically characterize mutants for the trait of interest. In this regard, mutant populations are desirable as the mutations are typically superimposed on to a uniform genetic background. Thus, any nucleotide variation observed between lines is predicted to be caused by treatment with mutagen and not the result of natural nucleotide variation. The most commonly used chemical mutagen used in plants is Ethyl methane sulfonate (EMS)). This mutagen works preferentially on guanine resulting in G to A and C to T transitions. The density of induced mutations is estimated between 2–10 mutations/Mb in a diploid genome [27]. Known frequencies of mutation induced by a mutagen may help in predicting size of the population required for targeting specific genes [28]. Development of a novel genetic resource using mutagenesis is extremely useful in discovering new genes as well as for establishing their function. For example, the role of mutant alleles of different genes including GPC-1 [29], SBEII [30] and VRN [31] have been described using wheat mutated populations.

In the present study, a mutagenized wheat population is developed using EMS. Disease resistant as well as susceptible mutants are identified. These stable mutants are exposed to exome capture assay for identifying alleles conferring resistance to rust diseases. Through in silico evaluation of identified putative mutations, we recovered one SNP (out of 104,779 SNPs) in the R gene family (*Lr21* gene) that may have functional impact on resistance to rust diseases. The generated information would be useful in developing disease resistant wheat cultivars.

## Materials and methods

### Plant material

The hexaploid ‘NN-Gandum-1’ (*Triticum aestivum* L) is a spring wheat variety that was bred by making a cross (Chirya-3/Opata//2x parula/3/ Rohtas-90) at the PGMB Labs, NIBGE, Faisalabad Pakistan. ‘NN-Gandum-1’ (NN-1) has been evaluated for studying its adaptability throughout the Punjab province in Pakistan. Because of its high yield potential and limited tolerance to rust diseases (moderately resistant) especially leaf rust, this variety was selected for exposing to a mutagen. In this regard, seed were exposed to chemical mutagen ethyl methane sulphonate (EMS).

### Mutagenesis and mutant population development

To optimize the dose of EMS, 24 batches of 50 seed each were treated with different EMS concentrations (ranging from 0.4–0.9%); each concentration was also tested for two different time exposure and temperatures i.e. 1 and 2 hours and 33°C and 35°C, respectively. After treatment, seed were washed under running water for 3 hours to remove any residual EMS. Before the treatment, seed were sterilized with 5% and 70% sodium hypochlorite and ethanol, respectively, each with three washings. On the basis of germination percentage (45–55%), 0.8% EMS for 2 hour at 35°C was chosen to mutagenize a batch of 7,500 wheat seed.

**Fig 1:**
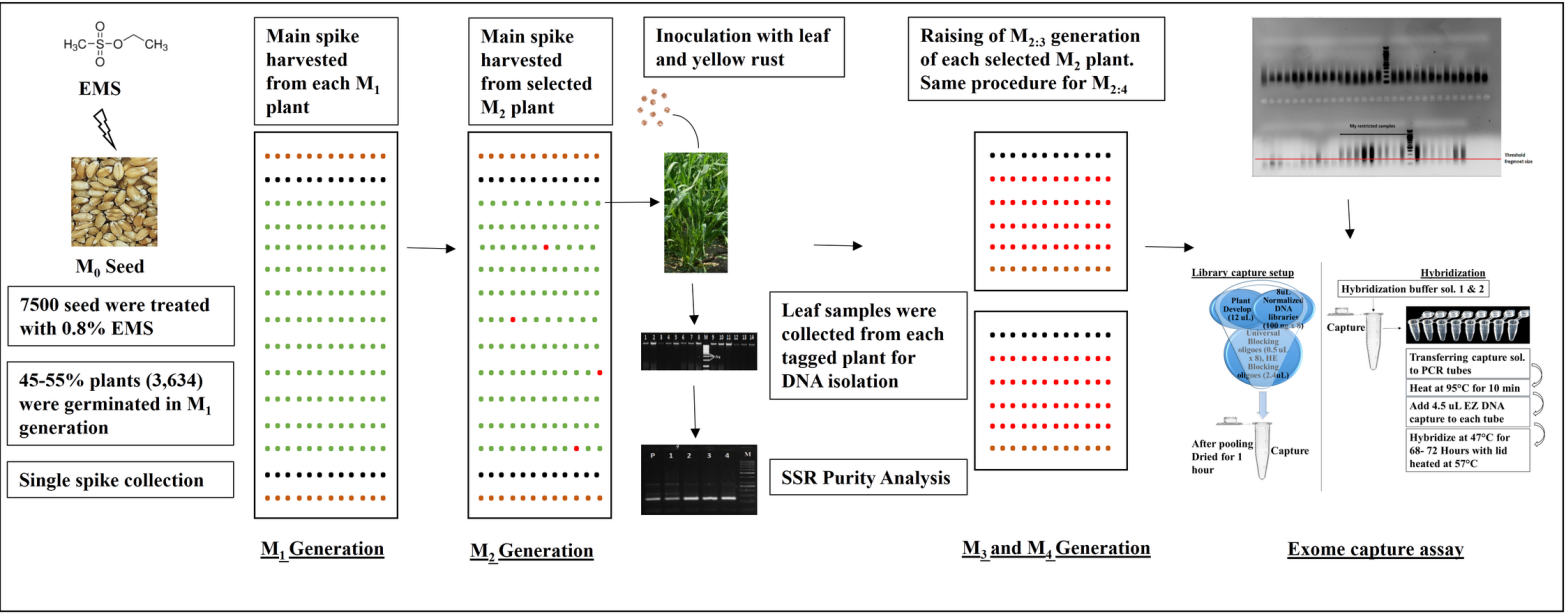
Development and screening of the wheat mutant population. Brown color dots represent the genotype ‘Morocco’, Black color dot represent ‘NN-Gandum-1’ (wild type), Green dots represent mutant plants, Red represents the selected mutant plants.

After an overnight drying, M_1_ seed were manually sown in experimental field in NIBGE (Faisalabad, Pakistan) with a cultivation distance of 30 cm between each row. Seed were sown in the form of beds, each bed contains 100 rows and each row contains eight plants. In total 3,634 M_1_ (out of 7,500 seed) plants were germinated. At physiological maturity, the main spike of each M_1_ plant was bagged to ensure self-pollination. At maturity, the M_2_ seed from each main spike was harvested to sow M_2_ generation. Standard agronomic practices were applied from sowing till harvesting in each generation. Eight M_2_ plants (of each M_1_ plant) in each row were sown in bed and were thinned to leave a single M_2_ plant per row. At key development stages, from germination to maturity, based on a visual characterization of plant, a systemic phenotyping scoring of the mutant population was carried out. Response to rust diseases (yellow and brown rust) was recorded by adopting a universally used rating scale [32, 33]. The coefficient of infection (CI) was calculated by multiplying the disease response value with the disease infection intensity. Average coefficient (ACI) was also calculated out by summing CI values of each genotype divided by the total number of locations. Leaf tissues were collected from each labeled plant per row for DNA isolation (CTAB method) for conducting SSR analysis (DNA uniformity analysis). Plant showing natural variations were rejected. Plants were advanced to M_3_ by adopting the same procedure as done for the M_2_. The main selfed spike was sown to develop the M_4_ population. Data for morphological traits as well as response to rust diseases were recorded. Out of these, 11 mutant lines showing strong resistance (nine mutant lines) and high susceptibility (two mutant lines) were selected for the exome capture assay (Fig 1).

### SSR uniformity analysis

In total, 20 SSRs were selected based on their high PIC values—calculated by surveying a set of wheat genotypes (96) with 650 SSR (unpublished results), and were surveyed on M_2_ plants. Out of these, WMS-46, WMS-249 and WMS-311 amplified polymorphic alleles.

### Preparation of wheat exome capture libraries

#### Exome capture design

Experiments for capturing exons were conducted by adopting the following protocols and procedures.

#### Isolation of genomic DNA and initial preparation for exome capture

Total genomic DNA was extracted from young seedlings of M_4_ plants of 12 samples (11 mutants and one wild type (non-mutagenized plant of the parental genotype) by adopting a large-scale extraction protocol [34] with few modifications. Initially, the nuclei were purified followed by treatment with proteinase K and purification with phenol-chloroform. The extracted genomic DNA concentration was normalized to a final concentration of 200 ng/μL by adding a 0.1 X TE buffer (0.1 mM EDTA, 10mM Tris-HCl, pH 8) [34]. In total 130 μl final volume of each sample was made.

The genomic DNA from each sample was fragmented to an average size of 250–450 bp using a Covaris E220 ultra sonicator (Duty cycle = 20%, intensity = 175 W, cycles per burst = 200 and time = 90 sec). The ends of the resultant fragments were repaired by adding 2.5 μL end repair enzyme followed by the ligation of A-tails and adaptors using KAPA’s kit and Bioo Adapters (Sciclone G3 robot). Genomic libraries were constructed for undertaking exome capture experiment using high-throughput library preparation kits manufactured by KAPA Biosystems, Inc. (Wilmington, MA, USA, catalog number KK2612). This procedure was described by Krasileva and co-workers [12].

#### Capture setup

In total, 1.2 μg of genomic DNA libraries were prepared by adding 150 ng of each of the eight samples. In this tube, 12 μL Plant ‘Developer’ reagent from Nimblegen (catalog number 6684335001) was added. In this solution, 0.5 μL barcode HE blocking oligoes (Bioo, catalog number 520999) and 2.4 μL HE universal blocking oligo (Bioo) were also added. After pooling all these components, the capture was dried in a vacuum centrifuge with heat for one hour. To facilitate this process, a hole was made in the lid of reaction tube. After drying, the hole was sealed [12].

#### DNA preparation for hybridization

After capture preparation, each capture was dissolved in 7.5 μL hybridization solution-5 and 3 μL hybridization solution-6 (Nimblegen hybridization kit (5634253001, Roche)). This was vortexed vigorously for 10 seconds and centrifuged shortly to collect the solution. The solution was transferred to a pre-labeled 0.2 μL PCR tubes. These tubes were heated at 95μC for 10 minutes in a thermo cycler. Next, a total of 4.5 μL of Nimblegen EZ DNA capture (custom, Roche) was added to each tube. The solution was mixed thoroughly by vortexing followed by pulse centrifugation to collect the solution [12].

#### Hybridization

This step was performed in a thermal cycler for 70 hours at 47°C while the lid temperature was kept at 57°C. Captured hybrids were washed using three washing buffer (wash buffer I, II and III) and one bead wash buffer [12].

### PCR amplification and final clean-up

Captures were amplified by PCR using primer pairs, F: AATGATACGGCGACCACCGATCTACAC and R: CAAGCAGAAGACGGCATACGAGAT (custom synthesized by Sigma). Master stock solution was prepared with 10 μM Tris (pH 8.0) at 100 μM, and the working solution was 10 μM (1:10 dilution of master stock) with 1:1 primer mix (5 μM of each primer). The PCR cocktail for two reactions was prepared by adding 20 μL captured DNA, 25 μL 2x KAPA mix and 5 μL primer stock (10 μM). PCR amplification was conducted in duplicates using KAPA’s amplification kit, and programmed for one cycle for 45 sec at 98°C followed by 10 cycles each for 15 sec at 98°C, 30 sec at 60°C and 30 sec at 72°C followed by a final extension cycle for 1 min at 72°C. The library was left on beads. The remaining 10 μL of the captured beads was kept for quality control checks at -20°C[12, 25]. The PCR products were purified by extensive washing (five times) using 80% ethanol (Sigma, catalog number ET0005) by adopting a published protocol [35].

#### DNA quantification

The enrichment of the targeted exons was tested using qRT-PCR. In this regard, the purified captured DNA was quantified, and primers designed for the amplification of two wheat housekeeping marker genes (F: ATCGGATTCGACAACATGC, R: ATATGGCCTGTCGTGAGTGA for Nuclear-encoded Rubisco and F: AAAGGCGTCAAGATGGAGTT, R: GGAATCCACCAACCATAACC for Malate Dehydrogenase) were custom synthesized by Sigma [12].

#### Illumina sequencing

The final captures were submitted to Beijing Genome Institute (BGI) for sequencing with Illumina Hisequation 2000 (Illumina).

#### Alignment of exome sequencing reads against wheat draft genome

**Fig 2:**
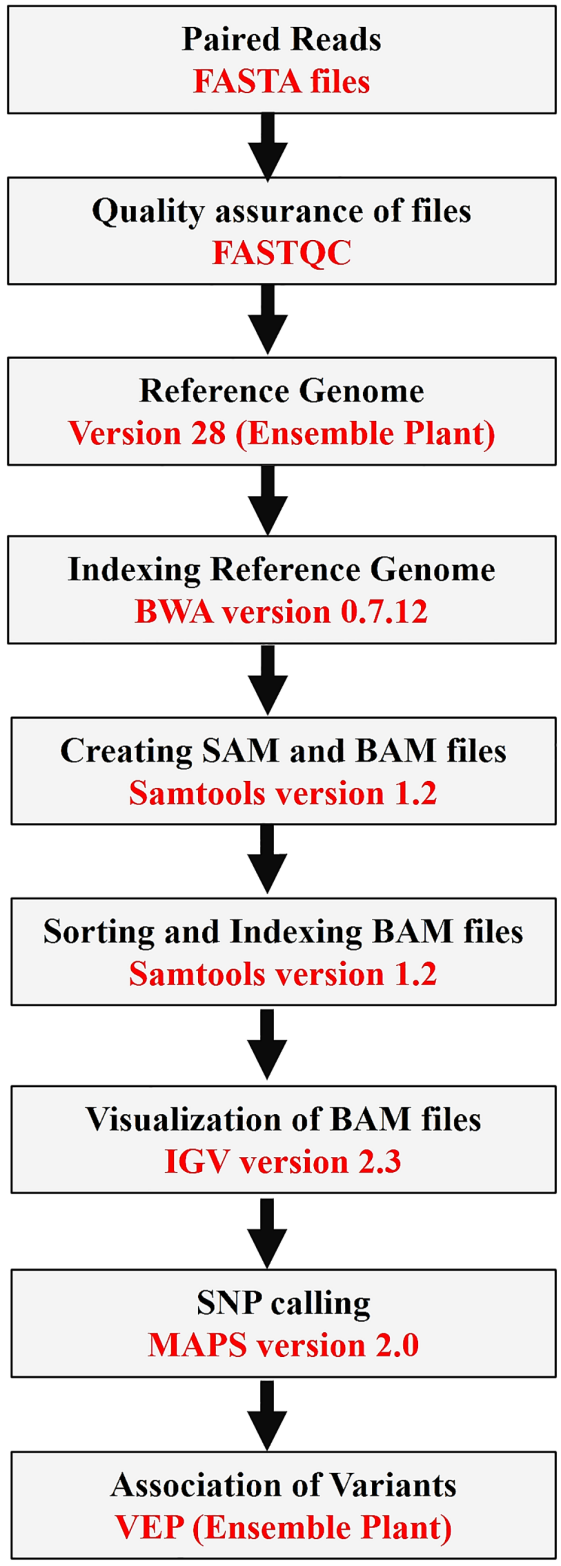
Schematic diagram showing various steps deployed for the identification of mutations.

Bioinformatics analysis of sequence of each fragment was undertaken from start to stop codon. Illumina generated-sequences were processed using Fastq software for testing the quality of reads. The generated reads were assembled and aligned against wheat reference genome using ‘bwa aln’ and ‘bwa sampe’ programs [36]. Then samtools and bamtools were used to generate sequence alignment map (SAM) and binary version of SAM files called as BAM file [37] (Fig 2).

#### Calling SNPs

Mutation and polymorphism survey (MAPS) (http://comailab.genomecenter.ucdavis.edu/index.php/MAPS) and ‘mpileup’ pipeline (http://comailab.genomecenter.ucdavis.edu/index.php/Mpileup) were deployed to select bases in the reference covered by at least one read at quality > 20 in a minimum number of samples. To differentiate the real SNPs from false mutations and or sequencing errors, an additional MAPS parameter was used. The threshold was setup independently for homozygous and heterozygous [12]. Mutation rate (frequency) was estimated by dividing the number of SNPs with the size of the exon of the corresponding chromosome [25]. Mutations were identified in all 11 mutant samples (N1-32, N1-61, N1-127, N1-236, N1-252, N1-506, N1-700, N1-701, N1-827, N1-910 and N1-1621) after aligning with the wheat draft genome (TGACv1) (http://plants.ensembl.org/Triticum_aestivum/Info/Index).

#### Effect of SNPs on gene

Mutations affecting gene function were identified by running a Variant Effect Predictor (VEP) from Ensembl tools release 78 in offline mode [38]. This software estimates the impact of SNPs including deletions, insertions, or structural variants on various genes, transcripts, and protein sequence, as well as on regulatory regions. The effects predicted by VEP were extracted and counted for determining the number of genes interrupted by stop/splice or missense mutations. Mutations affecting each gene were also counted. In some cases mutations were predicted to affect more than one gene. In these cases both effects were considered. If the same mutation found in more than one mutant line, it was counted multiple times. At the time the presented work was done, the wheat genome annotation was in its initial stages of development, thus some mutations identified by VEP as ‘intergenic’ could actually be in genes which were not yet completely annotated [39].

#### SNP analysis and validation

For the analysis and validation of DNA sequence polymorphisms and SNPs, DnaSP software was used. DnaSP is a multi-propose program that allows conducting exhaustive DNA polymorphism analysis using a graphical user-friendly interface.

## Results

### Population development and phenotyping

‘NN-Gandum-1 (NN-1)’ has been released for cultivation because of its high yield potential in Punjab, Pakistan. However, this cultivar showed limited tolerance to rust diseases (expressed as moderately resistant), especially leaf rust. For this reason we selected it for mutagenesis with the goal of improving disease resistance. Seed were collected from the seed center of PGMB, NIBGE Faisalabad for exposing to the chemical mutagen ethyl methane sulphonate (EMS).

EMS mutagenesis of NN-1 was optimized to determine LD50. After conducting a series of experiments using various concentrations of EMS as well as exposure time length, 0.8% (V/V) for 2 h at 35°C was found optimum. Following this, a total of 7,500 seeds were exposed to the mutagen, and were sown under the natural field conditions. In total 3,634 M_1_ seedlings were successfully emerged. The main spike of each plant was self-pollinated and harvested separately. In the following year, a single row of each M_1_ plant was sown to develop an M_2_ population. Plant #1 of each row was tagged for data collection and DNA extraction. Plants were scored for their response to rust diseases shown in Table 1. In total, 33 plants (0.91%) and 20 plants (0.55%) showed absolute resistance to leaf and yellow rust, respectively. While 126 plants (3.46%) and 127 plants (3.49%) exhibited high susceptibility to the leaf and yellow rust, respectively(S1 Fig).

**Table 1:** Response to rust disease showed by 3,634 M_4_ generation.

Sr. no.	Major categories	Phenotype Sub-categories	Scale	Parent	M_4_ Mutant generation	
					#	%
**1**	**Leaf Rust**	Resistant	R	60MR^a^	33	0.91
** **	** **	Mod. Resistant	MR		2366	65.12
** **	** **	Mod. Susceptible	MS		1109	30.52
** **	** **	Susceptible	S		126	3.46
**2**	**Yellow Rust**	Resistant	R	40MR^a^	20	0.55
** **	** **	Mod. Resistant	MR		2364	65.05
** **	** **	Mod. Susceptible	MS		1123	30.9
** **	** **	Susceptible	S		127	3.49

^a^Moderately Resistant

### Uniformity analysis

Before advancing the M_2_ plants, microsatellite (SSR) markers were surveyed to identify the true mutants generated through EMS versus natural variants. Natural variants, such as those arising from cross pollination between diverse cultivars are expected to show a high polymorphism rate when using SSRs. In total, 8.9% (natural variants) of the total M_2_ plants were rejected (unpublished data). The confirmed NN-1 derived mutants were advanced to M_4_ by self-pollination (Fig 1). Phenotypic data for each generation was recorded. A total of 18 M_3_ plants were selected (based on their response to rust diseases), and their M_4_ seed was harvested separately.

### Exome capture

A previously developed exome capture array design was used [12]. Genomic DNA from the 18 M_4_ seedlings was harvested, but seven mutants were excluded because of poor DNA quality. In total, 11 mutants were processed for exome capture assay. A single capture experiment was performed including mutants as well as the wild type (NN-1). Captures were multiplexed from 12 wheat lines and sequenced together on an Illumina lane. The resultant sequenced reads were processed together through the MAPS pipeline, with each sample serving as a control for others. Thus the mutation identified were present only in one of the samples [25]. The impact of these mutations on the corresponding genes was studied using VEP.

### Mutation detection and identification

In total, 53, 40, 39 and 41 SNPs were identified in mutant sample # N1-32, N1-61, N1-127 and N1-827, respectively. These 173 mutations were identified either in 3 and 5 prime UTR or up and down stream regions of gene, thus amino acids remained unaltered. These mutants were not considered for further analysis. Highest number of mutations were observed in coding regions of mutants, N1-236 (38,060 SNPs), N1-701 (3,019 SNPs) and N1-700 (25,713 SNPs). A modest number of mutations were recorded in mutant N1-252, N1-506, N1-910 and N1-1621 (Table 2). Mutations were identified, in 1,116 genes of all selected samples. Out of these, genes associated with resistance to rust were studied (S1 Table).

**Table 2:** SNPs distribution in various genomic regions of 11mutants of NN-Gandum-1.

Sample ID	N1-32	N1-61	N1-127	N1-236	N1-252	N1-506	N1-700	N1-701	N1-827	N1-910	N1-1621	# of SNPs
**Total SNPs**	53	40	39	38060	2233	2691	25713	30194	41	3582	2133	104,779
**Homozygous SNPs**	20	17	13	8418	695	906	7862	12514	14	1593	562	32, 614
**Heterozygous SNPS**	33	23	26	29534	1438	1784	17584	17837	21	2432	1453	72, 165
**GC/AT transitions**	24	19	17	10034	746	1108	6569	7872	19	972	866	28, 264
**Homozygous GC/AT transitions**	18	0	0	8165	563	15	42	57	0	5	494	9, 359
**Splice donor variant**	1.89%	0	0	0.07%	0.09%	2.94%	0.05%	0.19%	0	0.14%	0	68
**Splice acceptor variant**	1.89%	0	0	0.08%	0.04%	5.41%	0.08%	0.08%	0	0.06%	0.05%	74
**Stop gained**	0	0	0	0.28%	0.58%	2.04%	0.30%	0.04%	0	0.14%	0.56%	343
**Frame shift variant**	0	0	0	0	0	0	0	0.38%	0	0.31%	0	1
**Stop lost**	0	0	0	0.03%	0.04%	0	0.02%	0	0	0	0	25
**Start lost**	0	0	0	0.04%	0.04%	3.45%	0.02%	0.01%	0	0.08%	0	29
**Missense variant**	11.32%	20%	12.82%	14.09%	14.02%	3.00%	14.62%	0.02%	19.51%	0.06%	16.60%	15, 046
**Missense variant splice region variant**	0	0	0	0.16%	0.13%	6.51%	0.16%	14.02%	0	15.16%	0.09%	169
**Synonymous variant**	13.21%	7.5%	15.38%	14.95%	13.48%	2.58%	14.99%	0.15%	17.07%	0.17%	12.52%	15, 722
**Stop retained variant**	0	0	0	0.02%	0	0	0.03%	15.41%	0	14.71%	0	28
**Coding sequence variant**	0	0	0	0.02%	0	0	0.01%	0.04%	0	0	0	15
**5 prime UTR variant**	7.55%	2.5%	0	2.07%	2.96%	2.38%	2.01%	0.02%	2.44%	0.00%	3.47%	2, 268
**3 prime UTR variant**	1.89%	0%	2.56%	3.48%	3.94%	2.39%	3.40%	2.21%	2.44%	2.74%	3.00%	3, 761
**Non coding transcript exon variant**	0	0	0	0.94%	0.58%	1.54%	0.93%	3.94%	0	3.55%	0.09%	716
**Intron variant**	11.32%	7.5%	17.95%	17.71%	16.03%	2.37%	17.02%	0.28%	14.63%	0.31%	15.56%	18, 378
**Upstream gene variant**	13.21%	15%	10.26%	12.10%	15.09%	2.44%	12.27%	18.10%	0.00%	18.17%	15.28%	12, 231
**Downstream gene variant**	13.21%	20%	17.95%	16.48%	16.08%	2.46%	16.24%	10.50%	26.83%	9.02%	15.05%	17, 695
**Intergenic variant**	24.53%	27.5%	23.08%	16.10%	15.32%	2.68%	16.25%	18.23%	12.20%	16.64%	16.17%	16, 538
**Splice region variant**	0%	0%	0	1.41%	1.57%	2.45%	1.59%	14.78%	4.88%	16.81%	1.55%	1, 672

**Fig 3:**
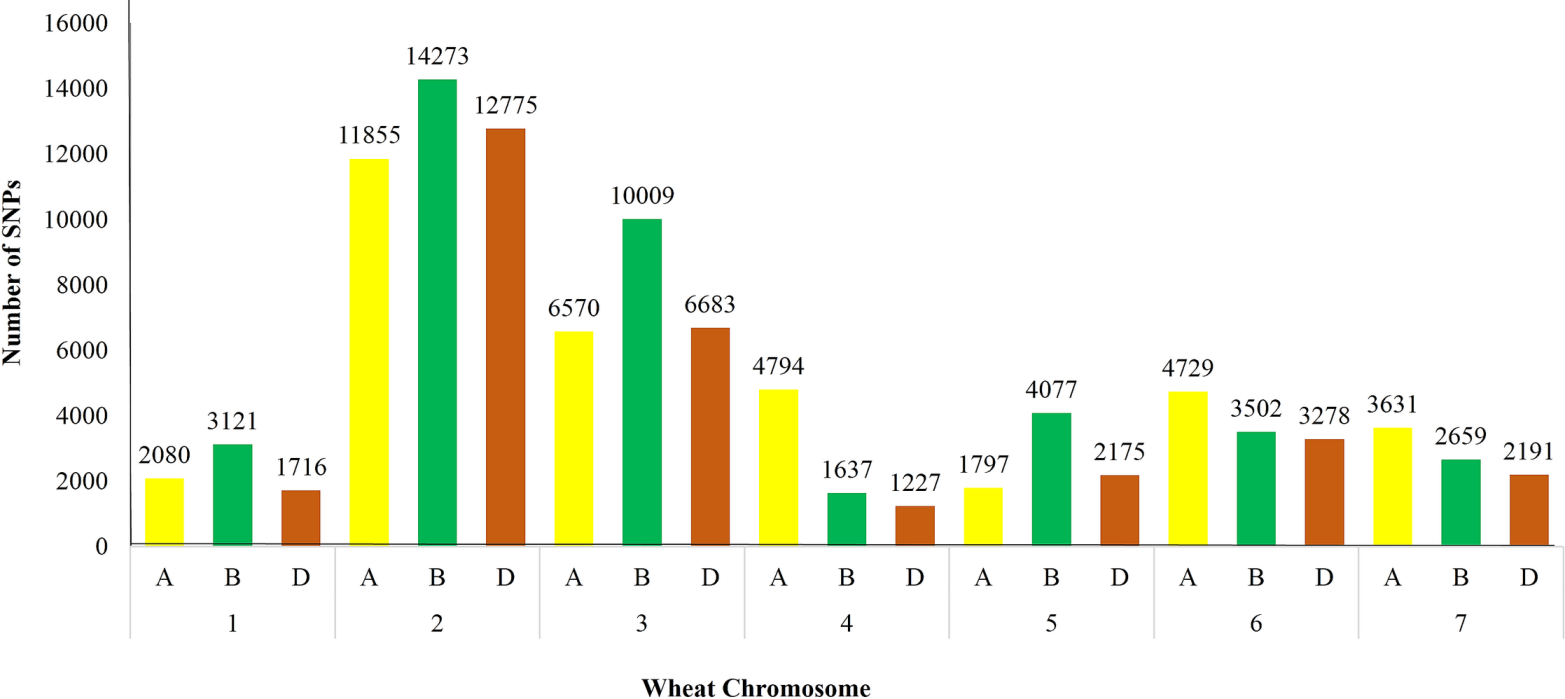
**Distribution of SNPs on various wheat chromosomes as well genomes (A, B, D)**.

**Fig 4:**
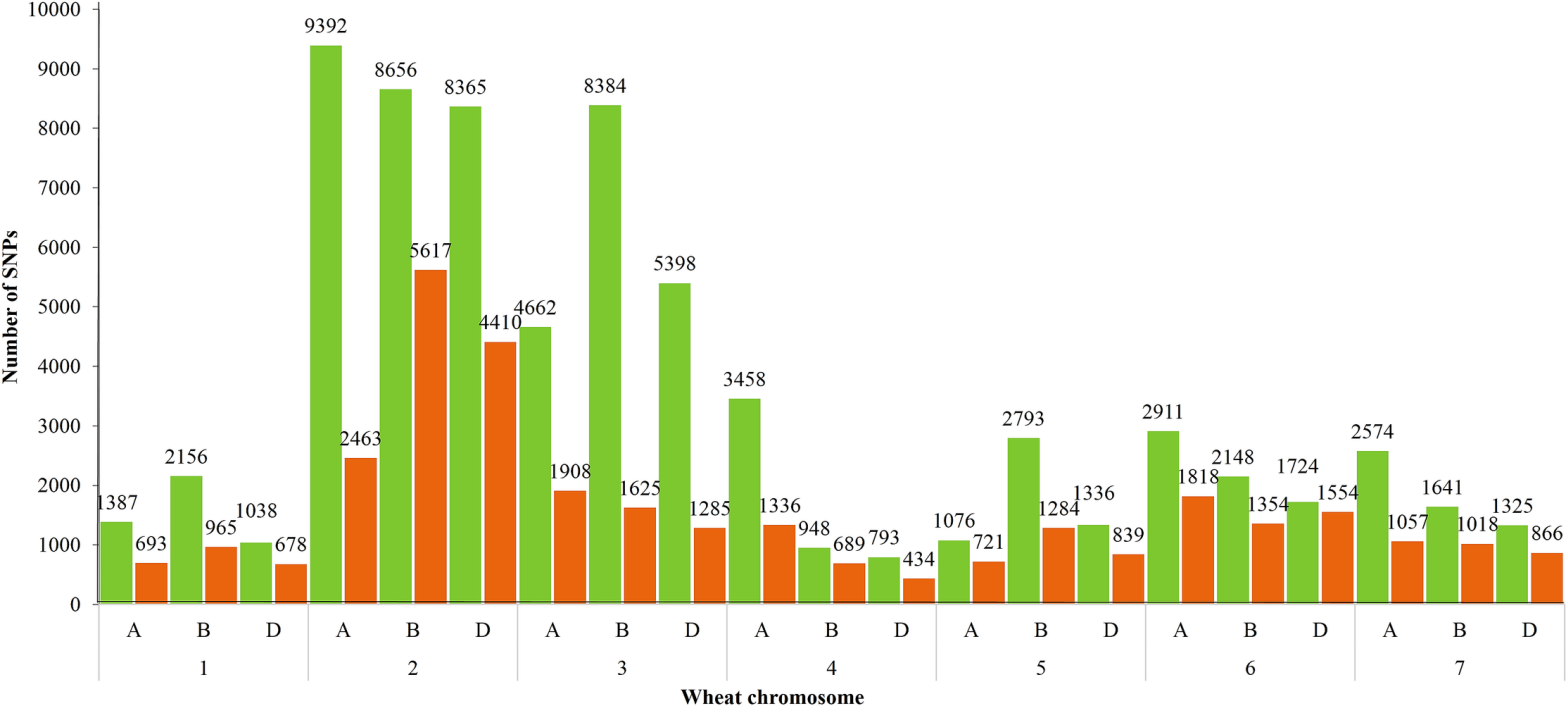
Occurrence of homozygous and heterozygous SNPs in genomes and chromosomes. The green column is representing heterozygous SNPs while red column is depicting homozygous SNPs.

**Fig 5:**
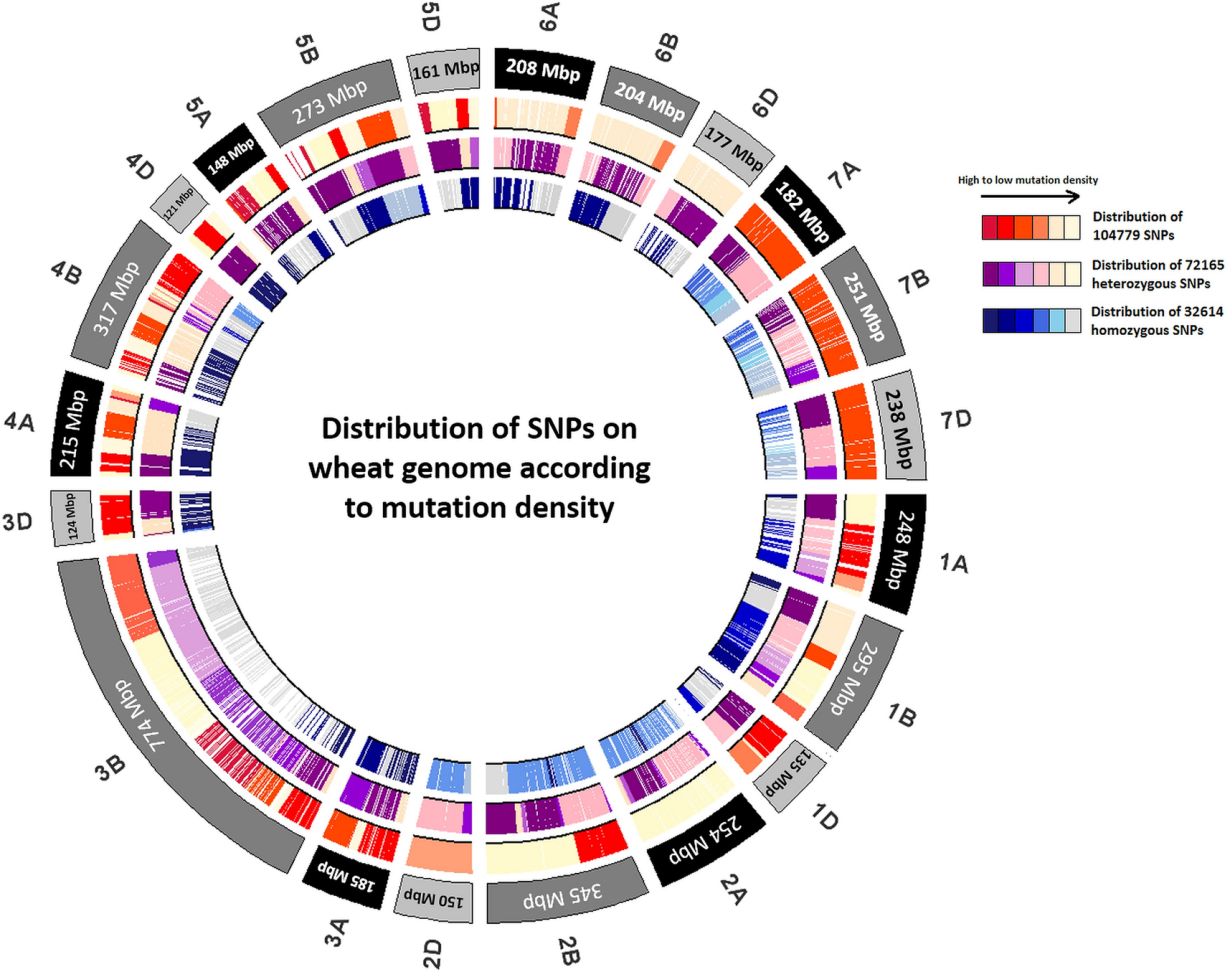
Circos plot: Distribution of SNPs according to mutation density. Light shade of the color represents low mutation density; Dark shade represents high mutation density.

The maximum number of SNPs (heterozygous as well as homozygous) were found in chr.2B (14273 SNPs) while the lowest number of SNPs were detected in chr.4D (1227 SNPs) (Fig 3). The highest and lowest number of homozygous mutations were observed in chr.2B (5617 SNPs) and chr.4D (434 SNPs), respectively (Fig 4). Distribution of all SNPs on different chromosomes is shown in circos plot (Fig 5). Owing to the fact that the total number of base pairs included in the assay varies by chromosome, we next evaluated the density of mutations. The highest mutation densities were observed in chr.3A, 3D, 7A, 7B and 7D. While the lowest mutation density was observed in chr.2A and chr.6D (Fig 5). The mean mutation rate was 14.4 mutations/Mb for heterozygous mutations and 6.51 mutations/Mb for homozygous mutations. In total, mean mutation rate was 20.91 mutations/Mb. The highest mutation rate (frequency) for the whole population was found in chr.2D (85.17 mutations/Mb) and lowest number of induced mutation rate was observed in chr.7D (2.83 mutations/Mb) (Table 3).

**Table 3:** Mutation frequency per Mb.

Chromosome	Size of sequenced exon in each chromosome (Mb)	SNPs	(No of mutations per Mb)	Mutation rate/kb
**1A**	248	2080	8.39	1 mutation /119.2 kb
**1B**	295	3121	10.58	1/94.5
**1D**	135	1716	12.71	1/78.7
**2A**	255	11855	46.49	1/21.5
**2B**	345	14273	41.37	1/24.2
**2D**	150	12775	85.17	1/11.7
**3A**	185	6570	35.51	1/28.2
**3B**	124	10009	80.72	1/12.4
**3D**	216	6683	30.94	1/32.3
**4A**	317	4794	15.12	1/66.1
**4B**	121	1637	13.53	1/73.9
**4D**	148	1227	8.29	1/120.6
**5A**	274	1797	6.56	1/152.4
**5B**	162	4077	25.17	1/39.7
**5D**	208	2175	10.46	1/95.6
**6A**	204	4729	23.18	1/43.1
**6B**	177	3502	19.79	1/50.5
**6D**	182	3278	18.01	1/55.5
**7A**	252	3631	14.41	1/69.4
**7B**	238	2659	11.17	1/89.5
**7D**	774	2191	2.83	1/353.4
**Heterozygous mutation**	5010	72165	14.40	
**Homozygous mutation**	5010	32614	6.51	
**Total**	5010	104779	20.91	

**Fig 6:**
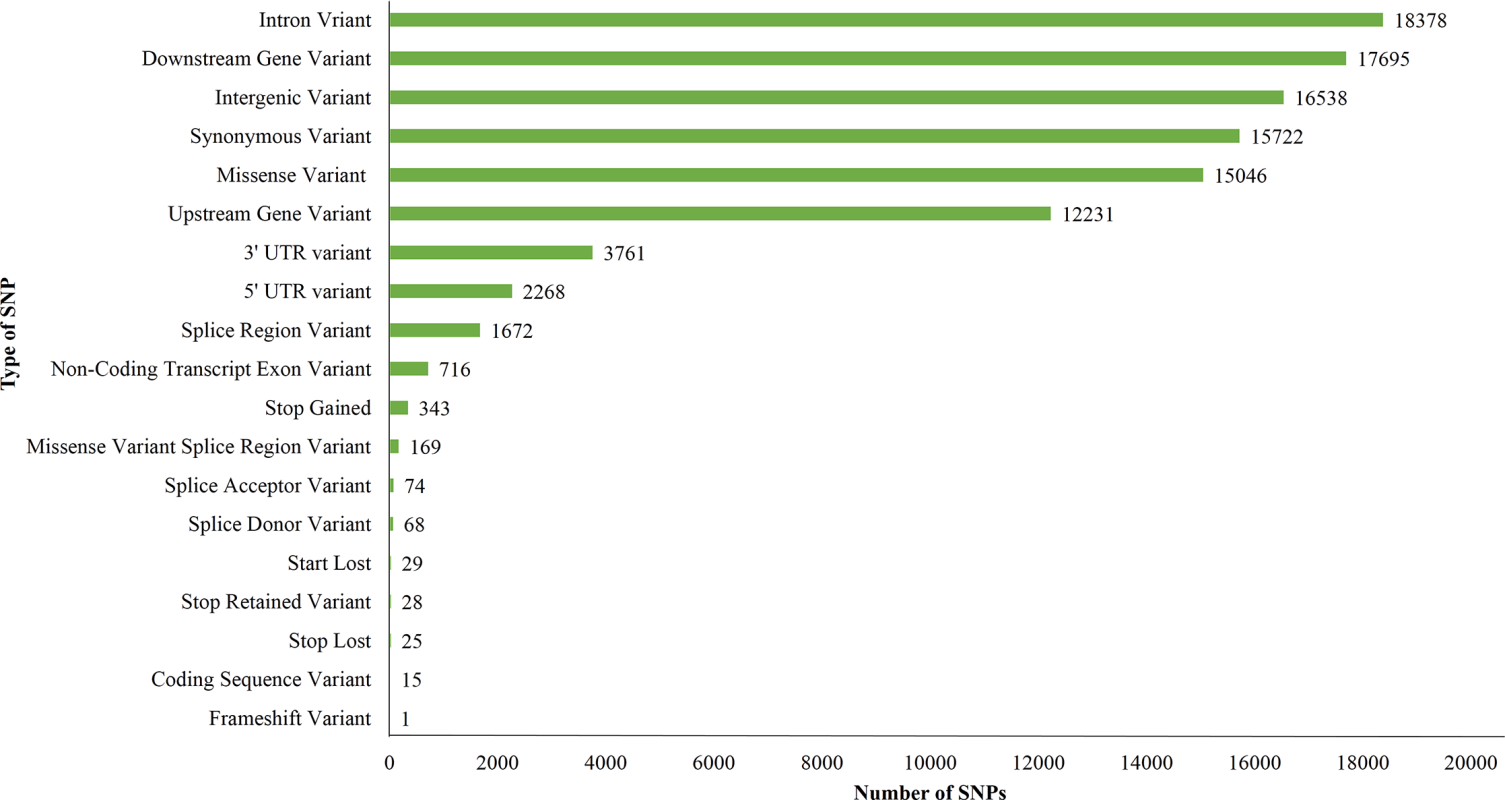
Type and number of mutations in 11 mutant lines.

In the present study, SNPs found in introns, non-coding transcript exons, upstream regions, downstream regions and intergenic regions were 17.54%, 0.68%, 11.67%, 16.88%, 15.78%, respectively. Similarly, mutations recorded in 3`UTR region, 5`UTR region, splice region, splice acceptor region and splice donor region were 3.58%, 2.16%, 1.6%, 0.07% and 0.06%, respectively. In total, 15% synonymous and 14.36% non-synonymous mutations were identified. While, in total 396 (0.38%) mutations were observed in stop codons including 343 stop gained, 28 stop retained and 25 stop lost mutations. However, 29 mutations resulted in ‘start lost’ in 18 genes (Fig 6).

Mutations found in genomic regions showing no impact on functional diversity were not considered for advancing the present investigations. In this context, heterozygous mutations were also ignored and homozygous mutations were considered. Out of the total homozygous SNPs (32,614), GC/AT transition mutations (6,723) were selected for further studies.These mutations were further filtered to 888 SNPs by selecting missense, stop/ start codon, splice region and frame shift variants. Out of these 888 variants, 91% were missense mutations. While 6.8%, 2.09% and 0.11% mutations were identified in stop/start codon, splice region, and frame shift variants, respectively. Out of these 888 mutations, 101 SNPs were found in genes conferring agronomically important traits including *Lr21* (disease resistance gene from R gene family), heat shock protein (*Hsp*), photosystem I (*PsaN*), micronutrients (potassium transporters and magnesium chelatase ChlI domain, PT and MCD), Armadillo-like helical complex gene (AHCG) and mitochondrial transcription factors (MTF) were selected for further study (Table 4).

**Table 4:** SNPs detected in example genes.

Gene	Gene Function	Chromosome	Position	Transition	Sample ID	Protein position	Amino acid	Codon	Gene ID	Exon	Reported variations	References
**Lr21**	Involved in resistance	1BS	3.4 Mb	A/C	N1-252	544	E/A	gAg/gCg	TRIAE_CS42_1BS_TGACv1_049811_AA0161950	3	55	[65, 71]
**hsp90**	Known for tolerance to heat, as well as tolerance to cold, UV light and healing of tissues after injury	4BS	14.57Mb	G/A	N1-236	319	A/V	gCc/gTc	TRIAE_CS42_4BS_TGACv1_328914_AA1095570	2	266	[72]
		1DS	0.29 Mb	G/A	N1-506	341	G/D	gGc/gAc	TRIAE_CS42_U_TGACv1_642737_AA2122480	9	1	[73]
		3DL	99.62 Mb	G/A	N1-700	138	A/T	Gca/Aca	TRIAE_CS42_3DL_TGACv1_252484_AA0890240	2	0	Not found
		4AS	48.97 Mb	G/A	N1-701	38	G/S	Ggc/Agc	TRIAE_CS42_4AS_TGACv1_306924_AA1015080	5	64	[74]
**PsaN**	Play role in photosynthesis	2BS	5.2 Mb	G/A	N1-700	96	A/T	Gcg/Acg	TRIAE_CS42_2BS_TGACv1_147121_AA0478340	3	32	Not found
**PT and MCD**	Role in channeling intermediates into the (bacterio) chlorophyll branch	5AL	10.7 Mb	G/A	N1-1621	235	S/N	aGt/aAt	TRIAE_CS42_5AL_TGACv1_377177_AA1244710	15	204	Not found
**AHCG**	Role in developmental and physiological processes	3AL	179.46	+3CAT/C	N1-701	875–877	3H/P	CATCATCAT/C	TRIAE_CS42_3AL_TGACv1_194056_AA0625580	20	212	[65]
**MTF**	Role for plant MTERFs^a^in response to abiotic stress	2DL	131.65 Mb	G/A	N1-701	249	E/K	Gag/Aag	TRIAE_CS42_2DL_TGACv1_158494_AA0520190	2	96	[75]
		3DS	18.43 Mb	G/A	N1-236	120	G/D	gGt/gAt	TRIAE_CS42_3DS_TGACv1_272327_AA0919100	4	89	
		6BL	4.32 Mb	G/A	N1-1621	2	A/T	Gca/Aca	Traes_6BS_1E0D70258	1	0	

^a^Mitochondrial transcription termination factors

One mutation was observed in *Lr21* (rust resistant) located in chr.1BS at 3.4 Mb position (mutant # N1-252) (Table 4, S2 Fig). The impact of this mutation on function of the mutant gene was studied by constructing the protein structure using 3D modeling. (S3 Fig). This mutation in *Lr21* converts glutamic acid (E) to alanine (A) at a position 544 AA. SNP in Lr21 was visualized using SnaSP software indicating the variation in gene against wild type (S4 Fig).

Similarly, four mutations were observed in *Hsp*gene. These mutations converts glycine to aspartic acid in chr.1DS at 0.29 Mb position, alanine to threonine in chr.3DL at 99.62 Mb position, glycine to serine in chr.4AS at 48.97 Mb position and alanine to valine in chr.4BS at position 14.57 Mb in mutants N1-506, N1-700, N1-701 and N1-236, respectively. Likewise, single mutations were observed in *PsaN*(mutant N1-700), magnesium chelatase ChlI domain (mutant N1-1621) and Armadillo-like helical complex gene (mutant N1-701). These mutations convert alanine to threonine, serine to asparagine and three histidine to one proline in chr.2BS, chr.5AL, and chr.3AL at position 5.2 Mb, 10.7 Mb and 179.46 Mb, respectively. Also, a total of three mutations were recorded in mitochondrial transcription factors in chr.2DL, chr.3DS and chr.6BL (mutant N1-700, N1-701 and N1-1621, respectively) (http://plants.ensembl.org/index.html) (Table 4). These mutations convert glutamic acid to lysine, glycine to aspartic acid and alanine to threonine at 131.65 Mb, 18.43 Mb and 4.32 Mb position, respectively.

## Discussion

In this study, the use of molecular and computational methods is described to identify induced mutations throughout the genome of hexaploid wheat var ‘NN-Gandum-1’. Before conducting exome capture, a mutant population was developed by exposing seed of NN-Gandum-1 to EMS (0.8% v/v) for 2 hours at 35°C. In previous studies, different doses of EMS were used for mutagenizing wheat seed, i.e. 0.6% to 1.0%. Fluctuations in dose concentration are largely dependent upon the exposure time and genetic makeup of the seed [40]. In the present study, a modest population size (3,634 M_1_) was assessed to obtain mutations in most genes of the wheat genome. In previous reports, varying population sizes (from 1,536 to 6,066) were used for identifying mutations in hexaploid wheat genome [41–44]. Population sizes can be increased to increase the chances of retrieving multiple deleterious alleles in all genes. However, handling a large size population is extremely difficult. Such massive size of the population can be achieved by planting M_1_ seed in batches in different wheat growing seasons.

We measured a range of traits (plant height, growth habit, days to heading, spikes length and response to rust diseases (brown and yellow rust) in a mutagenized population of NN-1 grown in the field for three generations (M_2_–M_4_). Only a small fraction of M_2_ plants were found resistant to rust diseases (0.91% for leaf rust and 0.55% for yellow rust). Most plants of the two mutant populations expressed phenotypes identical to their parent genotypes and only small number of plants (0.5%) expressed abnormal phenotypes [45]. In a different mutant population, the cumulative frequency of visual mutations was recorded as 20–37% for various traits in Cadenza [42]. Such fluctuations in frequency of mutant phenotypes with varying frequency for each trait were also found in our study. Comprehensive studies were conducted on cereals including barley and rice. In barley, 30–33% of the population developed through EMS and sodium azide expressed visibly different phenotypes than the wild type [46, 47]. Rice mutant population developed by exposing to N-methyl-N-nitrosourea (MNU) exhibited a very high percentage (50%) of visible mutant phenotypes. Out of these, many plants showed multiple abnormalities [48]. In sunflower, approximately 5% of the mutant population depicted altered morphologies than that of the wild-type [49]. Many commonalities in frequency of visible phenotypes of mutagenized populations of barley, rice and sunflower were reported [42, 50]. This data highlights the complexity of using visual phenotypes to estimate the efficiency mutagenesis. Contributing factors to phenotypic variation likely include the ploidy of the species, genotype used, mutagen dose and the environment in which screening for the traits is undertaken [51]. For example, effectiveness of EMS was more pronounced on sesame (*Sesamum indicum L*.) than that of γ rays [52]. In chickpea, the effectiveness and efficiency of EMS were seven and two times higher than that of γ rays [53]. In another study, effectiveness of hydrazine hydrates (HZ) towards inducing mutations in quantitative traits was higher than by the γ rays but less than the EMS [54]. However, in another study, similar effectiveness by exposing with of both the mutagens were found on *Stevia rebaudiana* [55].

Mutation frequency and density are largely dependent upon three factors including frequency of primary changes in genomic DNA, probability of repair and recognition (https://genetics.knoji.com/three-factors-affecting-mutation-rate/) by the host machinery. However, owing to the randomness of chemical mutagenesis and the fact that most mutations will be functionally silent, it is expected that the number of mutations accumulating in different regions of the genome will fluctuate. Indeed, it was demonstrated that mutation density ranged from 1–20 mutations per Mb among different individuals even if the same mutagen with the same concentration is applied [25]. In the present study, the mean mutation density was 20.91 mutations/Mb. These fluctuations in mutations density occurred largely due to differential permeability of EMS in seeds, mutagenesis, and the capacity of the cell to repair the genomic DNA. In multiple studies, fluctuations in average mutation density (19.6 mutations/Mb, 20.1 mutations/Mb, 23 mutations/ Mb and 33 mutations/ Mb) were found for wheat [12, 25, 40].

In present study, the highest mutation density was detected in chr.2D (one mutation per 11.7 kb). Without further data it is difficult to speculate on the nature of this observation. Larger scale exome capture studies in EMS mutagenized populations of hexaploid wheat where more than 6 million mutations were analyzed suggest that chr. 2D has a higher mutation density than that some chromosomes, but not notably higher than others such as chr.5D (12). Mutation densities fluctuate between EMS treatments and our observations may simply reflect variations unique to our particular population. Higher densities of mutations can accumulate in genomic regions where the potential of deleterious alleles created by EMS is lower. This is likely to be less pronounced in polyploids. Missense changes with a predicted moderate effect on gene function predominate for chr.2D (S2 Table). Previously, fixed deletions were detected at the Xcs1Vrga1-2A, KpnI -2B, and -2D loci in wheat [56]. In another report, there was evidence of inversion in chr. 2D in *Ae*. *Tauschii* [57].

In wheat, a total of 121 mutations including silent, missense and knockout were identified in waxy genes by surveying 2,348 EMS-treated M_2_ plants [58]. In another study, high mutation densities in hexaploid (one mutation per 38 kb) as well as tetraploid (one mutation per 51 kb) wheat populations were found [38]. Similarly, a total of 67 SNPs in four genes (*LBP*,-*COMT1*, *HCT2*, and *4CL1*) were identified. In waxy genes, mutation frequency was one SNP per 17.6 to34.4 kb in polyploid wheat while one SNP per 90 kb was found in *T*. *monococcum* TILLING population [59]. In another investigation, 246 alleles of the waxy genes were identified by exploring 1,920 mutants of both allohexaploid and allotetraploid wheat [60]. A total of 464 high-confidence SNPs were identified across the three mutagenized lines with mutation rate of ~35 SNPs per Mb [26]. Also, >10 million mutations in protein-coding regions of 2,735 mutant lines of tetraploid and hexaploid wheat (with an average of 30–40 mutations per kb) were found. Cumulatively, 23–24 missense and truncation alleles per gene, with at least one truncation or deleterious missense mutation in more than 90% of the captured wheat genes per population, were found [12]. In a segregating wheat EMS population, a clear peak region on 4-BS chromosome associated with increased plant height was identified, however, SNP showing impact on the mutant phenotype was not identified [61]. Such variations in mutation rate were reported in other crops also. In rice, while surveying for mutations in more than 38,000 independent M_4_ lines, 0.01% to 0.10% mutations were found [62]. In another study, a total of 27 and 30 SNPs were identified in rice mutant populations developed by exposing with EMS and a combination of sodium azide with methyl-nitrosourea, respectively [27]. Suzuki and co-workers (2008) calculated 7.4 to 10.6 mutations per 135 kb [48] in M_2_ rice mutant population. In another investigation, 18,000 induced mutations in 72 independent M_2_ rice plants were identified. Out of these, potentially deleterious mutations in >2600 genes were probed [25]. In sorghum, five mutations (one mutation per 526 kb) were identified [63]. Likewise in barley, in total, 382 mutations were detected in 182.2 Mb. The average mutation density was one mutation per 477 kb. The majority of mutations were G/C to A/T transitions, while ~8% were transversions. Missense mutations (61% of the total) were largely found in coding regions while the remaining were silent (37.5%) and nonsense mutations (1.1%) [64].

Given the variation in mutation densities reported elsewhere and the large variation in selected mutant lines of this study, it is intriguing to consider strategies to improve mutagenesis. This becomes reasonable once the cost of sequencing-based approaches is reduced sufficiently to consider routine application on many lines. For example, one can consider a mutant population with a narrow distribution of mutation density where all plants harbor the maximal amount of induced mutations. This would provide the highest efficiency of mutation recovery. However, maximizing density may incur fitness penalties such that some alleles will not accumulate. Alternatively once could consider a strategy whereby the average mutation density of the population is suitable, but the distribution of mutation densities of individual lines is broad. It is interesting to note that lines used in the present study were pre-selected by phenotype, yet show a broad distribution of mutation density. Another important consideration with regards to mutation density is the effect of background mutations when trying to unambiguously assign the mutation that is causing the observed phenotype. As mutation density increases, so to do the number of background truncation mutations and other predicted deleterious changes that can confound the selection of candidate genes. This is especially an issue when induced mutations are genetically linked.

Previous studies demonstrated that effective discovery of mutations in mutant wheat lines is possible irrespective of the fact that using hexaploid wheat genome as a reference hinders the ability, if not largely, for discriminating the homoeologous genes [25]. The MAPS software used in the study ensures the identification of true-to-type mutations by comparing sequence changes for each of the sampled position in many samples, high transition rate of GC to AT and no mutations in wild type [25]. In the present study, a limited number of samples (11 mutants and one wild) were compared to avoid any loss of rare mutations as well as managing the computational load. Once identified, putative mutations can then be evaluated for their effect on gene function. The VEP software was used which can analyze, annotate, and prioritize variants in coding and non-coding parts of the genome. This software is user friendly in configuring and extending the analysis. It offers several other advantages including high reproducibility coupled with its inherent ability to interpret variants [39]. In the present study, 104,779 SNPs were filtered to 101 SNPs using filtration criteria. Mutations with the highest probability for causing deleterious impact were considered i.e. homozygous mutations (excluded heterozygous). Then, homozygous GC/AT transition mutations were selected, and out of these truncation mutations like stop codons and splice site changes, non-synonymous changes (missense mutation), predicted to be deleterious were considered. Only one frame shift mutation was found in one wheat mutant line (N1-701) in chr.3AL at position 179.46 Mb in Armadillo-like helical complex gene. This gene confers protein that is involved in different developmental and physiological processes in simple as well as multicellular eukaryotes [65].

The chimeric allele containing one mutation (C/T) in *Lr21* was identified. Upon translation to protein sequence it was found that glutamic acid has been replaced by alanine, and found in activated part of the protein. Previously, a total of 55 variations were reported in this gene(ID: TRIAE_CS42_1BS_TGACv1_049811_AA0161950). Most variations were detected in NBS domain (active region) and LRR regions of the gene (https://plants.ensembl.org).Earlier, a number of inactive *Lr21* alleles were reported in bread wheat which got activated by the intermolecular recombinations of two haplotypes [66]. This gene is 4318 bp long and encodes 1080 amino acid protein, contains conserved NBS domain as well as 13 imperfect LLRs and 151 amino acid stretch that is missed in NBS-LLRs proteins at N terminus [66]. In the present study, the mutation found in chimeric allele was located in exon-2 of NBS domain.

In field experiments, strong rust resistance was observed in mutant N1-252 compared to the wild type (S1 Fig). *Hsp* gene was also linked with rust resistance as the resistant-mediated signaling pathways require wheat homologs of heat shock protein (*HSP90*) [66–68]. Four mutations were observed in *Hsp* in chr.1DS, chr.3DL, chr.4AS and chr.4BS at position 0.29 Mb, 99.62 Mb, 48.97 Mb and 14.57 Mb in plant mutant N1-506, N1-700, N1-701 and N1-236, respectively. Previously 331 variations were reported in *Hsp* gene (http://plants.ensembl.org/index.html) (Table 4). Similarly, mutations were also identified in *PsaN*, micronutrients, Armadillo-like helical complex gene andmitochondrial transcription factor in chr.2BS, chr.5AL, chr.3AL, chr.2DL, chr.2DS, chr.3DS and chr.6BL (Table 4). In total 32, 204, 212 and 208 variations were reported previously in *PsaN*, micronutrients, Armadillo-like helical complex gene andmitochondrial transcription factor, respectively (http://plants.ensembl.org/index.html). Similarly, mutations in several genes including starch biosynthesis, fatty acid biosynthesis, flowering pathways genes, flowering time, waxy gene, *Rht-B1*, Opaque-1, male sterility *Ms2*, *FatA* (acyl-ACP thioesterase), *SAD* (the stearoyl-ACP desaturase), and amylose, resistant starch (*GBSSI-7A*, *GBSSI-4A*, *GBSSI-7D*, *SBEIIa*, *SBEIIb*) were reported in previous studies [12, 41–43, 69].

## Conclusions

Exome capture is found suitable in identifying mutations induced by EMS in hexaploid wheat with relatively big genome size (~17.6 Gb). Application of new sequencing approaches coupled with improved bioinformatics tools for processing and managing data (MAPS, VEP, etc.) made it possible to look for genuine mutations in the subset of the genome (the exome) where mutations have the highest change of affecting gene function. The size of plant exomes is relatively similar compared to the large fluctuations in overall genome size. Thus exome capture can be translated to other crops irrespective of the genome size, and costs should be comparable. In species where a reference genome is not yet available, a transcript genome assembly can be made at relatively low cost [70]. In the present study we show that induced mutations can be recovered in genes implicated in agronomically important traits. The study also sheds light on the distribution of mutation densities in lines within our population. From this work we conclude that our novel population of mutant wheat contains a suitable density of mutations for future use as both a forward and reverse-genetic resource. In addition, we conclude that the methods discussed in this manuscript can be applied for dissecting genetic pathways and developing novel phenotypes for breeding by design approaches to sustain wheat productivity in upcoming years.

## Supporting information

S1 Fig Response of various M_4_ plants to rust disease. (A) Attack of leaf and yellow rust on wild type (moderately resistant). (B) Mutant line # N1-252 (resistant to the disease), (C) Severe disease infection on mutant line # N1-910.
(TIF)

S2 Fig Diagrammatic view of ‘*Lr21*’ with number of exons and introns, and position of SNP. (TIF)

S3 Fig Protein modeling of *Lr21* gene for wild type and resistant mutant (#252). (TIF)

S4 Fig *Lr21* gene sequence comparison of mutant #252 with wild type. (TIF)

S1 Table Mutated genes with sequence variation. (DOCX)

S2 Table Mutation type and mutation in genes in chr.2D, and their impact on gene function. (DOCX)
