# Supplementary figures and images for: Identification of induced mutations in hexaploid wheat genome using exome capture assay
Source: PLoS One. 2018 Aug 13;13(8):e0201918. doi: 10.1371/journal.pone.0201918 (PMC6089429; doi:10.1371/journal.pone.0201918)

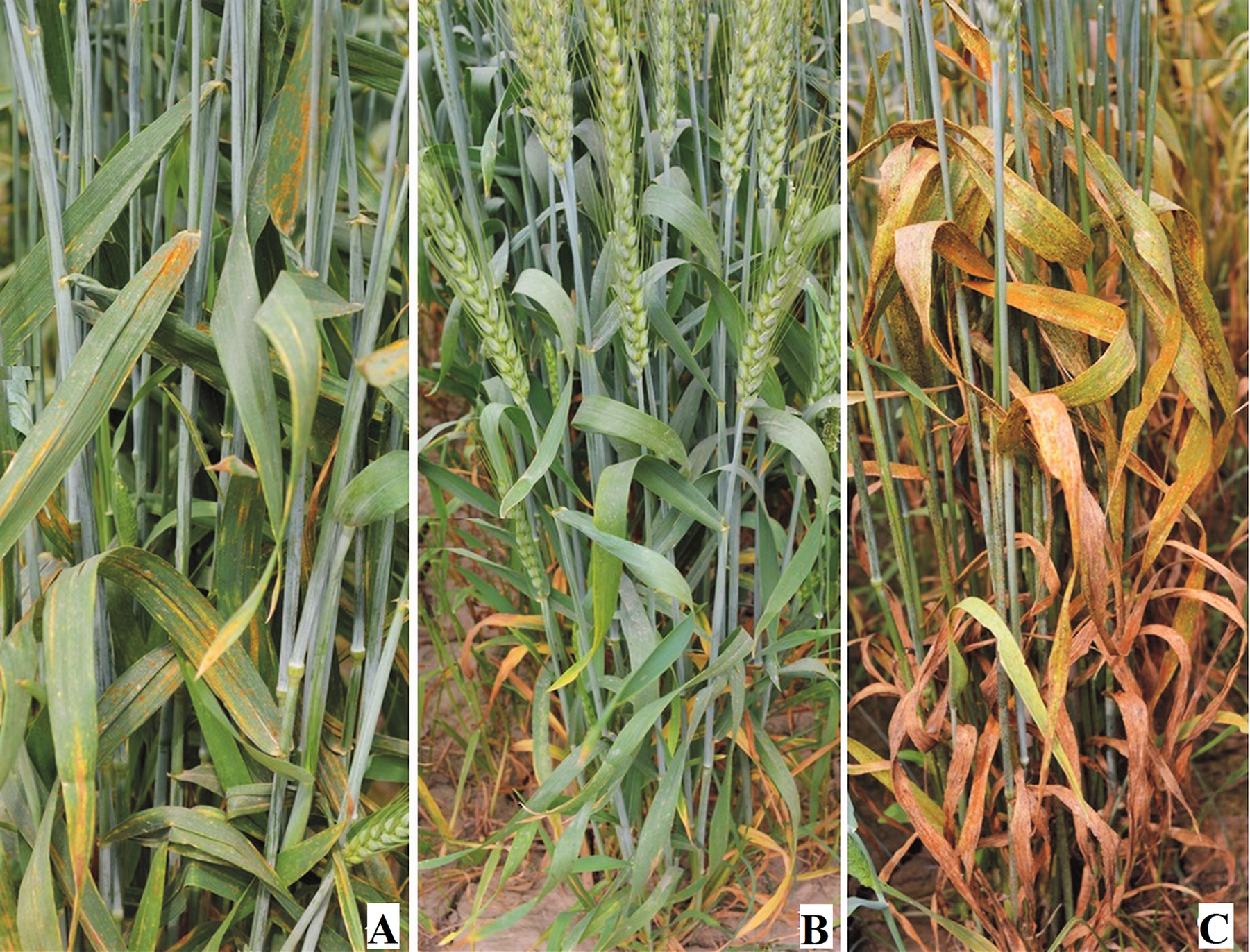

Supplement: S1 Fig — (A) Attack of leaf and yellow rust on wild type (moderately resistant). (B) Mutant line # N1-252 (resistant to the disease), (C) Severe disease infection on mutant line # N1-910. (TIF) [file pone.0201918.s001.tif]

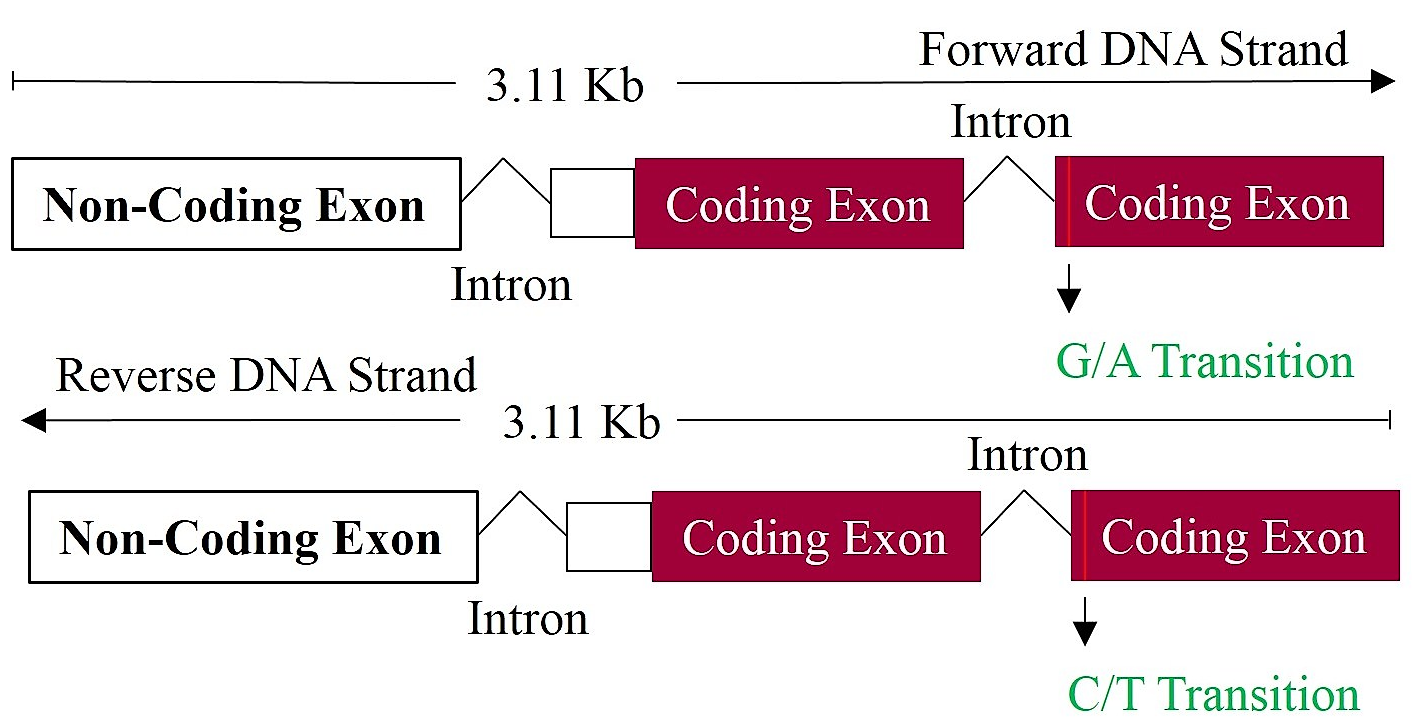

Supplement: S2 Fig — (TIF) [file pone.0201918.s002.tif]

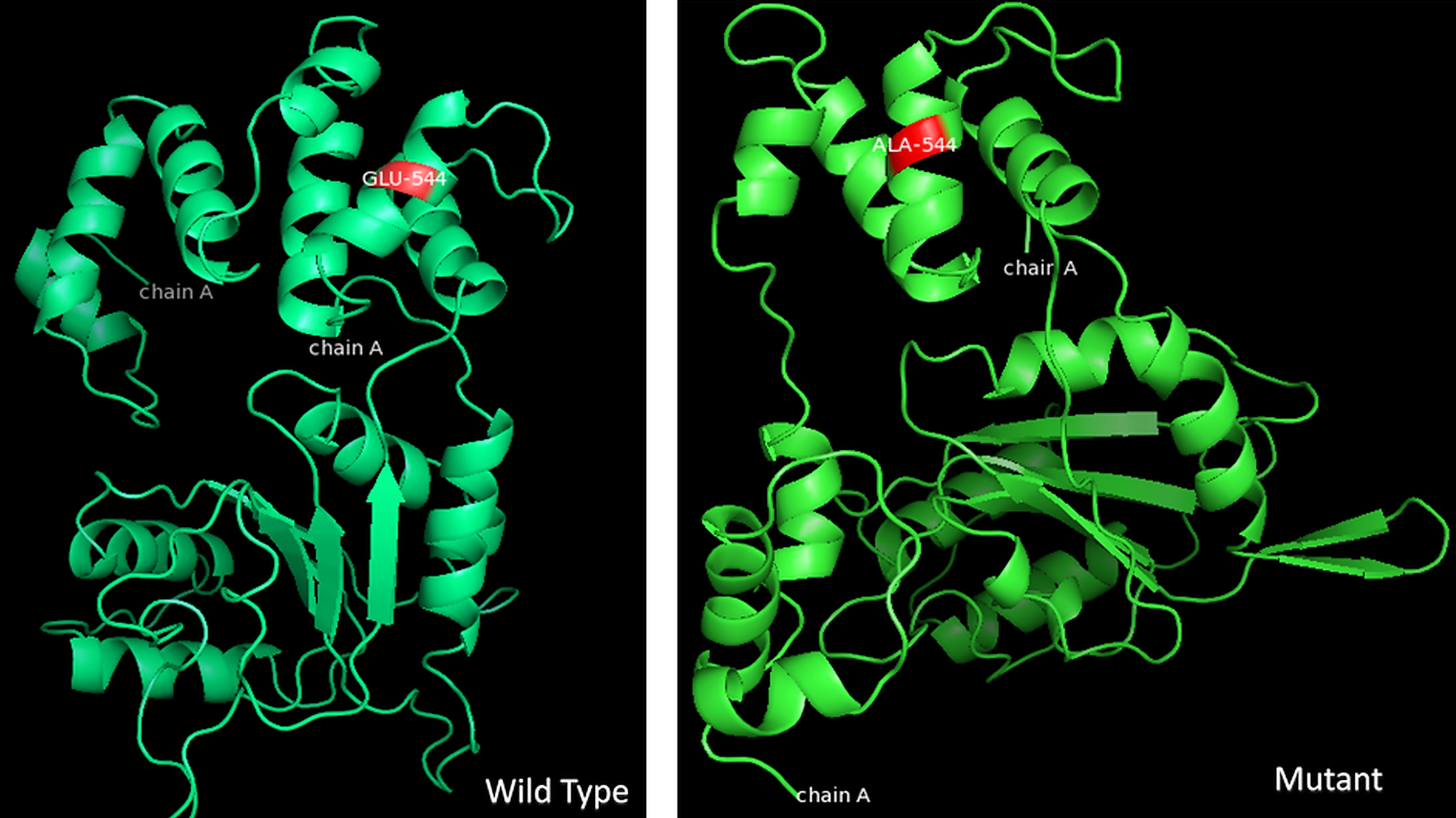

Supplement: S3 Fig — (TIF) [file pone.0201918.s003.tif]

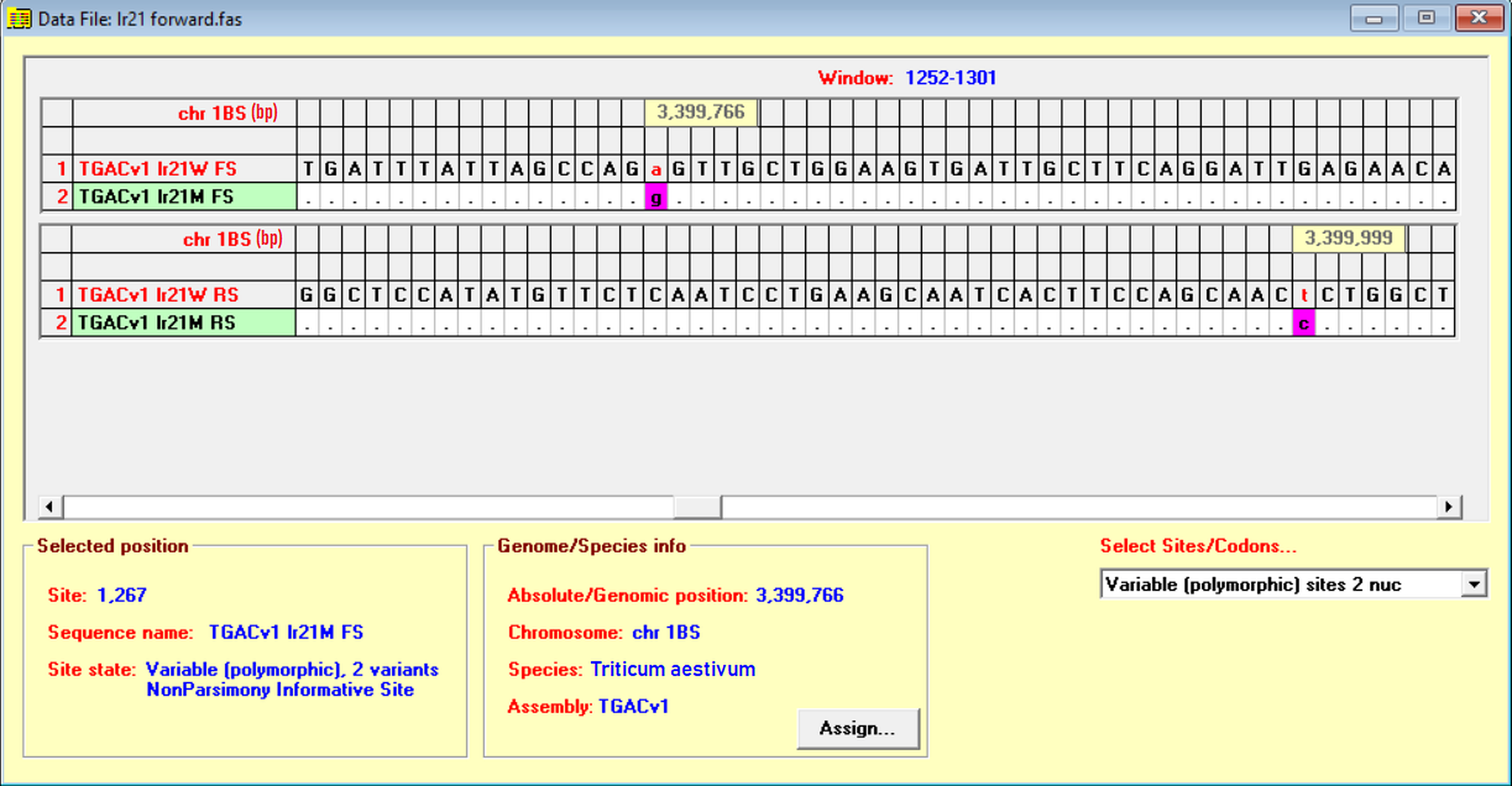

Supplement: S4 Fig — (TIF) [file pone.0201918.s004.tif]
