# Supplementary material for: Identification of induced mutations in hexaploid wheat genome using exome capture assay
Source: PLoS One. 2018 Aug 13;13(8):e0201918. doi: 10.1371/journal.pone.0201918 (PMC6089429; doi:10.1371/journal.pone.0201918)
Supplement: S1 Table — (DOCX) [file pone.0201918.s005.docx]

| **Sr. #** | **Sample ID** | **Response to Rust** | **# of mutated genes** | **Mutated genes explored for analysis** | **Sequence information** |
| --- | --- | --- | --- | --- | --- |
| **1** | N-32 | Resistant | 2 | NAC domain | Gtt/Att |
|  |  |  |  | UDP-glucuronosyl/UDP-glucosyltransferase | Gcc/Acc |
| **2** | N-61 | Resistant | 2 | Protein kinase domain, | aGc/aAc |
|  |  |  |  | Extracellular ligand-binding receptor | aGa/aAa |
| **3** | N-127* | Susceptible | - | - | - |
| **4** | N-236 | Resistant | 279 | Hsp | gCc/gTc |
|  |  |  |  | Mitochondrial transcription | gGt/gAt |
|  |  |  |  | Root cap | Gcc/Acc |
| **5** | N-252 | Resistant | 27 | Lr21, | gAg/gCa |
|  |  |  |  | Plant disease resistance response protein | gGc/gAc |
| **6** | N-506 | Resistant | 47 | Hsp | gGc/gAc |
| **7** | N-700 | Resistant | 268 | Hsp | Gca/Aca |
|  |  |  |  | PsaN | Gcg/Acg |
|  |  |  |  | Heavy metal-associated domain | Gtg/Atg |
| **8** | N-701 | Resistant | 412 | Hsp | Ggc/Agc |
|  |  |  |  | Armadillo-like | CATCATCAT/C |
|  |  |  |  | Mitochondrial transcription | Gag/Aag |
|  |  |  |  | Root cap | gGc/gAc |
| **9** | N-827* | Resistant | - | - | - |
| **10** | N-910 | Susceptible | 53 | Armadillo-like helical, | Gcc/Acc |
|  |  |  |  | Root cap | Gtg/Atg |
| **11** | N-1621 | Resistant | 26 | Micronutrients, | aGt/aAt |
|  |  |  |  | Mitochondrial transcription | Gca/Aca |

*Information is not given for sample # N-127 and N-827 because of low quality SNPs

**Title: Table S1. Mutated genes with sequence variation**
