# Supplementary material for: Identification of induced mutations in hexaploid wheat genome using exome capture assay
Source: PLoS One. 2018 Aug 13;13(8):e0201918. doi: 10.1371/journal.pone.0201918 (PMC6089429; doi:10.1371/journal.pone.0201918)
Supplement: S2 Table — (DOCX) [file pone.0201918.s006.docx]

**Table S2. Mutation type and mutation in genes in chr.2D, and their impact on gene function**

| **Chr.** | **Position (Mbs)** | **Mutation type** | **Gene** | **Impact** |
| --- | --- | --- | --- | --- |
| 2D | 1.34 | Missense variant | - | Impact=moderate; strand=1 |
| 2D | 3.24 | Missense variant | Oxoglutarate/iron-dependent dioxygenase | Impact=moderate; strand=-1 |
| 2D | 3.43 | Missense variant | Alpha/Beta hydrolase fold | Impact=moderate; strand=1 |
| 2D | 3.47 | Missense variant | Ribosomal protein S13-like, H2TH | Impact=moderate; strand=1 |
| 2D | 3.84 | Missense variant | UDP-glucuronosyl/UDP-glucosyltransferase | Impact=moderate; strand=1 |
| 2D | 3.84 | Missense variant | UDP-glucuronosyl/UDP-glucosyltransferase | Impact=moderate; strand=1 |
| 2D | 4.05 | Stop lost | Isopenicillin N synthase-like | Impact=high; strand=-1 |
| 2D | 4.74 | Missense variant | - | Impact=moderate; strand=-1 |
| 2D | 4.76 | Missense variant | Winged helix-turn-helix DNA-binding domain | Impact=moderate; strand=-1 |
| 2D | 4.78 | Missense variant | Isoprenoid synthase domain | Impact=moderate; strand=-1 |
| 2D | 4.82 | Missense variant | Rossmann-like alpha/beta/alpha sandwich fold | Impact=moderate; strand=-1 |
| 2D | 4.97 | Missense variant | Protein kinase domain | Impact=moderate; strand=1 |
| 2D | 4.97 | Missense variant | Protein kinase domain | Impact=moderate; strand=1 |
| 2D | 5.19 | Missense variant | S-adenosyl-L-methionine-dependent methyltransferase | Impact=moderate; strand=1 |
| 2D | 5.29 | Missense variant | S-adenosyl-L-methionine-dependent methyltransferase | Impact=moderate; strand=1 |
| 2D | 5.31 | Stop lost | Protein kinase-like domain | Impact=high; strand=1 |
| 2D | 5.34 | Stop gained | Phospholipase A2 domain | Impact=moderate; strand=1 |
| 2D | 5.35 | Missense variant | Tetratricopeptide-like helical domain | Impact=moderate; strand=-1 |
| 2D | 5.37 | Missense variant | - | Impact=moderate; strand=-1 |
| 2D | 5.37 | Missense variant | Chloramphenicol acetyltransferase-like domain | Impact=moderate; strand=-1 |
| 2D | 5.37 | Missense variant | Nucleotide-binding alpha-beta plait domain | Impact=moderate; strand=1 |
| 2D | 5.38 | Missense variant | Glycoside hydrolase, catalytic domain | Impact=moderate; strand=-1 |
| 2D | 5.39 | Missense variant | Major facilitator superfamily domain | Impact=moderate; strand=-1 |
| 2D | 5.39 | Stop gained | - | Impact=moderate; strand=1 |
| 2D | 9.74 | Missense variant | - | Impact=moderate; strand=-1 |
| 2D | 9.81 | Stop gained | Oligopeptide transporter, OPT superfamily | Impact=high; strand=-1 |
| 2D | 9.82 | Missense variant | Oligopeptide transporter, OPT superfamily | Impact=moderate; strand=-1 |
| 2D | 9.83 | Missense variant | - | Impact=moderate; strand=1 |
| 2D | 9.84 | Missense variant | Concanavalin A-like lectin/glucanase domain | Impact=moderate; strand=1 |
| 2D | 9.85 | Missense variant | Protein kinase domain | Impact=moderate; strand=-1 |
| 2D | 9.85 | Missense variant | Protein kinase domain | Impact=moderate; strand=-1 |
| 2D | 9.85 | Missense variant | Leucine-rich repeat | Impact=moderate; strand=-1 |
| 2D | 9.86 | Missense variant | - | Impact=moderate; strand=-1 |
| 2D | 9.86 | Missense variant | Bulb-type lectin domain | Impact=moderate; strand=1 |
| 2D | 9.86 | Missense variant | Bulb-type lectin domain | Impact=moderate; strand=1 |
| 2D | 9.86 | Missense variant | Pyridoxal phosphate-dependent transferase | Impact=moderate; strand=-1 |
| 2D | 9.86 | Missense variant | Pyridoxal phosphate-dependent transferase | Impact=moderate; strand=-1 |
| 2D | 9.87 | Missense variant | Protein kinase domain | Impact=moderate; strand=1 |
| 2D | 9.87 | Missense variant | NAD(P)-binding domain | Impact=moderate; strand=1 |
| 2D | 9.88 | Missense variant | Eama domain | Impact=moderate; strand=-1 |
| 2D | 9.88 | Missense variant | Glycoside hydrolase, family 16 | Impact=moderate; strand=1 |
| 2D | 9.88 | Missense variant | Glycoside hydrolase, family 16 | Impact=moderate; strand=1 |
| 2D | 9.90 | Missense variant | NAD(P)-binding domain | Impact=moderate; strand=-1 |
| 2D | 9.90 | Stop gained | Zinc finger, RING/FYVE/PHD-type | Impact=moderate; strand=1 |
| 2D | 9.91 | Missense variant | Isopenicillin N synthase-like | Impact=moderate; strand=-1 |
| 2D | 9.91 | Missense variant | Isopenicillin N synthase-like | Impact=moderate; strand=-1 |
| 2D | 9.91 | Missense variant | Protein kinase domain | Impact=moderate; strand=1 |
| 2D | 9.91 | Missense variant | HAD-like domain | Impact=moderate; strand=1 |
| 2D | 9.91 | Missense variant, splice region variant | HAD-like domain | Impact=moderate; strand=1 |
| 2D | 9.91 | Missense variant | Armadillo-like helical | Impact=moderate; strand=1 |
| 2D | 9.91 | Missense variant | Bacterial periplasmic spermidine/putrescine-binding protein | Impact=moderate; strand=1 |
| 2D | 9.91 | Missense variant | Major facilitator superfamily domain | Impact=moderate; strand=-1 |
| 2D | 11.82 | Missense variant | Protein of unknown function DUF3741 | Impact=moderate; strand=-1 |
| 2D | 13.45 | Missense variant | Protein kinase domain | Impact=moderate; strand=-1 |
| 2D | 17.20 | Missense variant | Nucleotide-binding alpha-beta plait domain | Impact=moderate; strand=1 |
| 2D | 20.22 | Missense variant | - | Impact=moderate; strand=-1 |
| 2D | 21.58 | Missense variant | CRC domain | Impact=moderate; strand=1 |
| 2D | 21.91 | Missense variant | Protein of unknown function DUF599 | Impact=moderate; strand=1 |
| 2D | 24.71 | Missense variant | Isopenicillin N synthase-like | Impact=moderate; strand=-1 |
| 2D | 32.52 | Missense variant | Aldolase-type TIM barrel | Impact=moderate; strand=1 |
| 2D | 34.38 | Missense variant | Potassium transporter | Impact=moderate; strand=-1 |
| 2D | 35.29 | Missense variant | Lipopolysaccharide-modifying protein | Impact=moderate; strand=1 |
| 2D | 35.79 | Missense variant | Cation transporter | Impact=moderate; strand=1 |
| 2D | 35.79 | Missense variant | Cation transporter | Impact=moderate; strand=1 |
| 2D | 40.30 | Stop gained | Cullin, N-terminal | Impact=high; strand=-1 |
| 2D | 41.48 | Missense variant | NAD(P)-binding domain | Impact=moderate; strand=-1 |
| 2D | 47.25 | Stop gained, splice region variant | Mitochodrial transcription termination factor | Impact=high; strand=-1 |
| 2D | 49.06 | Missense variant | Tetratricopeptide-like helical domain | Impact=moderate; strand=-1 |
| 2D | 103.91 | Missense variant | Alpha/Beta hydrolase fold | Impact=moderate; strand=-1 |
| 2D | 105.69 | Missense variant | P-loop containing nucleoside triphosphate hydrolase | Impact=moderate; strand=1 |
| 2D | 105.88 | Missense variant | Multicopper oxidase, type 2 | Impact=moderate; strand=-1 |
| 2D | 107.03 | Missense variant | S-adenosyl-L-methionine-dependent methyltransferase | Impact=moderate; strand=-1 |
| 2D | 107.06 | Missense variant | - | Impact=moderate; strand=-1 |
| 2D | 107.52 | Missense variant | Protein kinase domain | Impact=moderate; strand=1 |
| 2D | 107.52 | Missense variant | Protein kinase domain | Impact=moderate; strand=1 |
| 2D | 108.49 | Missense variant | NAC domain | Impact=moderate; strand=-1 |
| 2D | 110.42 | Missense variant | Tetratricopeptide-like helical domain | Impact=moderate; strand=1 |
| 2D | 113.91 | Missense variant | - | Impact=moderate; strand=1 |
| 2D | 114.50 | Missense variant | ABC transporter type 1, transmembrane domain | Impact=moderate; strand=1 |
| 2D | 114.91 | Missense variant | Zinc finger, RING/FYVE/PHD-type | Impact=moderate; strand=1 |
| 2D | 124.26 | Missense variant | Calcineurin-like phosphoesterase domain, apah type | Impact=moderate; strand=1 |
| 2D | 127.97 | Missense variant | Thioredoxin-like fold | Impact=moderate; strand=1 |
| 2D | 129.71 | Missense variant | Ubiquitin-related domain | Impact=moderate; strand=1 |
| 2D | 129.87 | Missense variant | Tetratricopeptide-like helical domain | Impact=moderate; strand=1 |
| 2D | 129.95 | Missense variant | Protein of unknown function DUF716 (TMEM45) | Impact=moderate; strand=1 |
| 2D | 129.95 | Missense variant | Protein of unknown function DUF716 (TMEM45) | Impact=moderate; strand=1 |
| 2D | 130.06 | Missense variant | Glycoside hydrolase, family 28 | Impact=moderate; strand=-1 |
| 2D | 130.68 | Missense variant | Protein of unknown function DUF760 | Impact=moderate; strand=1 |
| 2D | 130.84 | Missense variant | Haem peroxidase, plant/fungal/bacterial | Impact=moderate; strand=-1 |
| 2D | 131.05 | Stop retained variant | START-like domain | Impact=low; strand=1 |
| 2D | 131.48 | Missense variant | Glycosyl transferase, family 1 | Impact=moderate; strand=-1 |
| 2D | 131.65 | Missense variant | Mitochodrial transcription termination factor | Impact=moderate; strand=1 |
| 2D | 131.65 | Missense variant | Mitochodrial transcription termination factor | Impact=moderate; strand=1 |
| 2D | 132.55 | Missense variant | Domain of unknown function DUF4220 | Impact=moderate; strand=1 |
| 2D | 132.69 | Stop gained | - | Impact=high; strand=1 |
| 2D | 132.69 | Missense variant | - | Impact=moderate; strand=1 |
| 2D | 132.72 | Missense variant | Protein kinase domain | Impact=moderate; strand=1 |
| 2D | 132.72 | Missense variant | Protein kinase domain | Impact=moderate; strand=1 |
| 2D | 133.63 | Missense variant | - | Impact=moderate; strand=1 |
| 2D | 133.79 | Missense variant | Rubisco LSMT, substrate-binding domain | Impact=moderate; strand=1 |
| 2D | 133.80 | Missense variant | - | Impact=moderate; strand=1 |
| 2D | 133.80 | Missense variant | - | Impact=moderate; strand=1 |
| 2D | 133.82 | Missense variant | Pentatricopeptide repeat | Impact=moderate; strand=-1 |
| 2D | 134.08 | Missense variant | - | Impact=moderate; strand=1 |
| 2D | 134.12 | Missense variant | Post-SET domain | Impact=moderate; strand=-1 |
| 2D | 134.12 | Missense variant | Post-SET domain | Impact=moderate; strand=-1 |
| 2D | 134.47 | Missense variant | NAD(P)-binding domain | Impact=moderate; strand=-1 |
| 2D | 134.57 | Missense variant | WD40 repeat | Impact=moderate; strand=1 |
| 2D | 134.78 | Stop gained | P-loop containing nucleoside triphosphate hydrolase | Impact=moderate; strand=-1 |
| 2D | 136.47 | Missense variant | Plastid lipid-associated protein/fibrillin conserved domain | Impact=moderate; strand=1 |
| 2D | 136.54 | Missense variant | Pathogenic type III effector avirulence factor Avr cleavage site | Impact=moderate; strand=1 |
| 2D | 136.58 | Missense variant | UHRF1-binding protein 1-like | Impact=moderate; strand=1 |
| 2D | 137.64 | Missense variant | V-type atpase, V0 complex, 116kda subunit family | Impact=moderate; strand=-1 |
| 2D | 137.66 | Missense variant | - | Impact=moderate; strand=1 |
| 2D | 138.16 | Missense variant | Major facilitator superfamily domain | Impact=moderate; strand=-1 |
| 2D | 139.24 | Missense variant | Mitochodrial transcription termination factor | Impact=moderate; strand=-1 |
| 2D | 139.37 | Missense variant | Nonaspanin (TM9SF) | Impact=moderate; strand=1 |
| 2D | 139.39 | Missense variant | Pentatricopeptide repeat | Impact=moderate; strand=1 |
| 2D | 139.39 | Missense variant | Pentatricopeptide repeat | Impact=moderate; strand=1 |
| 2D | 139.44 | Missense variant | Tetratricopeptide-like helical domain | Impact=moderate; strand=-1 |
| 2D | 139.67 | Missense variant | Ankyrin repeat | Impact=moderate; strand=-1 |
| 2D | 139.77 | Missense variant | - | Impact=moderate; strand=-1 |
| 2D | 140.17 | Missense variant | Jmjc domain | Impact=moderate; strand=1 |
| 2D | 140.19 | Missense variant | Cytochrome P450, conserved site | Impact=moderate; strand=1 |
| 2D | 140.19 | Missense variant | Cytochrome P450, conserved site | Impact=moderate; strand=1 |
| 2D | 141.52 | Missense variant | Zinc finger, RING/FYVE/PHD-type | Impact=moderate; strand=1 |
| 2D | 142.32 | Missense variant | Cytochrome P450, conserved site | Impact=moderate; strand=1 |
| 2D | 142.75 | Missense variant | Chloramphenicol acetyltransferase-like domain | Impact=moderate; strand=-1 |
| 2D | 142.75 | Missense variant | Chloramphenicol acetyltransferase-like domain | Impact=moderate; strand=-1 |
| 2D | 149.09 | Missense variant, splice region variant | Zinc finger, GATA-type | Impact=moderate; strand=1 |
| 2D | 149.47 | Missense variant | Leucine-rich repeat | Impact=moderate; strand=1 |
| 2D | 149.84 | Missense variant | Protein kinase-like domain | Impact=moderate; strand=-1 |
| 2D | 149.85 | Missense variant | Calcineurin-like phosphoesterase domain, apah type | Impact=moderate; strand=1 |
